# Predictive values of diagnostic codes for identifying serious hypocalcemia and dermatologic adverse events among women with postmenopausal osteoporosis in a commercial health plan database

**DOI:** 10.1186/s12913-018-3016-y

**Published:** 2018-04-10

**Authors:** Florence T. Wang, Fei Xue, Yan Ding, Eva Ng, Cathy W. Critchlow, David D. Dore

**Affiliations:** 1 Optum Epidemiology, 1325 Boylston Street, Suite 1000, Boston, MA 02215 USA; 2 Amgen Inc., One Amgen Center Drive, Thousand Oaks, CA 91320 USA; 3 Brown University School of Public Health, Providence, RI 02912 USA

**Keywords:** Administrative data, Dermatologic events, Hypocalcemia, Positive predictive value, Postmenopausal osteoporosis

## Abstract

**Background:**

Post-marketing safety studies of medicines often rely on administrative claims databases to identify adverse outcomes following drug exposure. Valid ascertainment of outcomes is essential for accurate results. We aim to quantify the validity of diagnostic codes for serious hypocalcemia and dermatologic adverse events from insurance claims data among women with postmenopausal osteoporosis (PMO).

**Methods:**

We identified potential cases of serious hypocalcemia and dermatologic events through ICD-9 diagnosis codes among women with PMO within claims from a large US healthcare insurer (June 2005-May 2010). A physician adjudicated potential hypocalcemic and dermatologic events identified from the primary position on emergency department (ED) or inpatient claims through medical record review. Positive predictive values (PPVs) and 95% confidence intervals (CIs) quantified the fraction of potential cases that were confirmed.

**Results:**

Among 165,729 patients with PMO, medical charts were obtained for 40 of 55 (73%) potential hypocalcemia cases; 16 were confirmed (PPV 40%, 95% CI 25–57%). The PPV was higher for ED than inpatient claims (82 vs. 24%). Among 265 potential dermatologic events (primarily urticaria or rash), we obtained 184 (69%) charts and confirmed 128 (PPV 70%, 95% CI 62–76%). The PPV was higher for ED than inpatient claims (77 vs. 39%).

**Conclusion:**

Diagnostic codes for hypocalcemia and dermatologic events may be sufficient to identify events giving rise to emergency care, but are less accurate for identifying events within hospitalizations.

**Electronic supplementary material:**

The online version of this article (10.1186/s12913-018-3016-y) contains supplementary material, which is available to authorized users.

## Background

Osteoporosis, a condition characterized by loss of bone mass and increased risk of fracture, affects approximately 10 million individuals in the United States (US) [1]. Approximately 30% of postmenopausal Caucasian women have osteoporosis; their lifetime fracture risk is estimated as 40% [2]. Treatment includes abaloparatide, bisphosphonates, calcitonin, denosumab, raloxifene, and teriparatide, some of which have been linked with increased risk of hypocalcemia and adverse dermatologic events [3–7]. Post-marketing safety studies of medicines often rely on administrative claims databases to identify adverse outcomes following drug exposure [8–11]. Valid ascertainment of outcomes is essential for accurate results. Yet, the accuracy of identifying hypocalcemia and adverse dermatologic events, especially the more serious events, using claims-identified codes is not well described.

We conducted a study to assess the validity of diagnostic codes in claims data for hypocalcemia and dermatologic events, as compared with medical record confirmation of events, among a population of women with postmenopausal osteoporosis (PMO).

## Methods

### Data source

This observational study was a retrospective analysis of medical and pharmacy claims data that are part of the Optum Research Database (ORD), a proprietary research database built from provider, facility, and pharmacy claims of a large US health insurer affiliated with Optum. The individuals covered by this health insurance are geographically diverse, and represent 3–4% of the US population.

There was no active enrollment or active follow-up of patients, and no data were directly collected from patients. The New England Institutional Review Board provided oversight during the conduct of this study and its Privacy Board granted a Waiver of Authorization for linkage of claims and medical records.

### Study population

The population included women with PMO who had medical and pharmacy coverage between June 2005 and May 2010. Women who were postmenopausal (age 55 years or older) and had diagnosis or treatment codes indicative of osteoporosis or osteoporotic fracture were eligible for the PMO population [12]. A list of relevant codes is provided in an Additional file 1: Table S1. We required at minimum 6 months of continuous enrollment in the health plan preceding the first code indicating PMO (baseline period). We used data from the baseline period to determine cohort eligibility and to characterize baseline attributes for study members.

### Identification of potential outcomes through claims data

Potential events were identified through diagnosis codes for hypocalcemia (ICD-9 275.41) or dermatologic adverse events (bullous dermatoses [ICD-9 694.xx], erythematous events [ICD-9 695.1×, 695.5×], or urticaria or rash [ICD-9 708.x, 782.1]) associated with an emergency department (ED) visit or inpatient hospitalization. To capture serious events, we excluded potential cases recorded outside of the ED or inpatient setting, and additionally included only events with codes recorded in the first (primary) position on the claim. Within our data system, the primary diagnostic code on hospitalization-associated claims represents the principal diagnosis, the condition established after study to be chiefly responsible for the admission [13].

### Case confirmation through medical record review

We sought medical records for all hypocalcemia, bullous dermatoses, and erythematous events and for a random sample of urticarial or rash events identified from the claims. A chronological list of claims for each of the potential cases was reviewed to determine the site of medical care most likely to yield the necessary information for case confirmation. A physician blinded to the patients’ osteoporosis medication use reviewed the medical records and classified each potential case as: definite case; definite non-case; or insufficient information. Definite cases of hypocalcemia were identified based on the designation of hypocalcemia diagnosis by either the admitting or consulting physician, with confirmation through the lab result that triggered the diagnosis. For both hypocalcemia and dermatologic events, a definite case classification also required attribution of the event as the leading cause for the hospitalization or ED visit (non-incidental cases).

### Statistical analysis

We calculated the positive predictive value (PPV) and associated 95% confidence interval (CI) among potential cases for which we obtained a medical record. The PPV was defined as the proportion of potential cases classified as definite cases—calculated overall, and for dermatologic events, by subgroups (bullous dermatoses, erythematous events, and urticaria/rash). CIs were calculated using the exact binomial Wilson method [14]. We stratified the results by site of care, and, in a secondary analysis, by provider specialty. To evaluate the importance of our requirement that events led to the hospitalization or ED visit, we included both non-incidental and incidental cases in the numerator of PPV estimates in a sensitivity analysis. In this case, incidental events were those confirmed events based on medical record review but listed within the medical record as a secondary reason for the hospitalization or ED visit.

## Results

The population consisted of 165,729 women with PMO, the majority of whom were between 55 and 64 years of age, and white, reflecting the underlying population of the database (Table 1). We sought charts for 55 patients with qualifying claims for hypocalcemia, for which 40 (73%) charts were received. Sixteen potential cases were confirmed hypocalcemia leading to hospitalization or ED visit, yielding a PPV of 40.0% (95% CI 24.9–56.7%) (Table 2). One potential case had insufficient information for adjudication. Claims associated with ED setting (PPV 81.8%, 95% CI 48.2–97.7%) performed better than those from inpatient setting (PPV 24.1%, 95% CI 10.3–43.5%). The PPV for hypocalcemia was higher for claims associated with emergency medicine (PPV 54.2%, 95% CI 32.8–74.4%) as compared with other provider specialties. The inclusion of incidental hypocalcemia events yielded a higher PPV (70.0%, 95% CI 54.6–81.9%).

**Table 1:** Baseline characteristics of PMO study population, June 2005 – May 2010

Characteristics	Number of patients (%)
	*N =* 165,729
Age (years)	
55 to 64	130,344 (78.6)
65 to 69	17,348 (10.5)
70 to 74	7966 (4.8)
≥ 75	10,071 (6.1)
Race	
Asian	3898 (2.4)
Caucasian	123,788 (74.7)
Hispanic	10,131 (6.1)
Black	12,579 (7.6)
Other	1200 (0.7)
Unknown	14,077 (8.5)
Geographic region	
Northeast	14,124 (8.5)
Midwest	35,746 (21.6)
South	98,519 (59.4)
West	16,965 (10.2)
Unknown	375 (0.2)
Calendar year of cohort entry	
2005	49,311 (29.8)
2006	34,899 (21.1)
2007	26,828 (16.2)
2008	28,508 (17.2)
2009	20,390 (12.3)
2010	5793 (3.5)
Usage of healthcare facilities	
Patients with at least one physician office/outpatient visit	154,648 (93.3)
Patients with at least one emergency room visit	28,381 (17.1)
Patients with at least one hospitalization	11,472 (6.9)

*Abbreviation*: *PMO* Post-Menopausal Osteoporosis

**Table 2:** PPV and 95% CI of serious hypocalcemia and serious dermatologic adverse event claims from emergency departments or inpatient facilities within the PMO study population

Event of interest	Charts requested	Charts obtained	Confirmed cases		
	N	N	*n*	PPV %^a^	95% CI^b^
Hypocalcemia					
Overall	55	40	16		(24.9–56.7)
By site of care					
Emergency department	12	11	9	81.8	(48.2–97.7)
Hospital	43	29	7	24.1	(10.3–43.5)
By provider specialty^c^					
Emergency medicine	31	24	13	54.2	(32.8–74.4)
Internal medicine	33	21	5	23.8	(8.2–47.2)
Cardiology	26	16	3	18.8	(4.0–45.6)
Other specialties^d^	44	30	8	26.7	(12.3–45.9)
With inclusion of incidental cases^e^	55	40	28	70.0	(54.6–81.9)
Dermatologic adverse events					
Overall	265^f^	184	128	69.6	(62.4–76.1)
Bullous dermatoses	6	3	1	33.3	(0.8–90.6)
Erythematous event	15	9	5	55.6	(21.2–86.3)
Urticaria or rash	247	173	122	70.5	(63.1–77.2)
By site of care					
Emergency department					
Overall	214	148	114	77.0	(69.4–83.5)
Bullous dermatoses	1	0	0	–	–
Erythematous event	7	3	2	66.7	(9.4–99.2)
Urticaria or rash	207	145	112	77.2	(69.5–83.8)
Hospital					
Overall	51	36	14	38.9	(23.1–56.5)
Bullous dermatoses	5	3	1	33.3	(0.8–90.6)
Erythematous event	8	6	3	50.0	(11.8–88.2)
Urticaria or rash	40	28	10	35.7	(18.6–55.9)
By provider specialty^c^					
Emergency medicine					
Overall	192	137	107	78.1	(70.2–84.7)
Bullous dermatoses	9	6	4	66.7	(22.3–95.7)
Erythematous event	3	2	1	50.0	(1.3–98.7)
Urticaria or rash	181	129	102	79.1	(71.0–85.7)
Internal medicine					
Overall	43	30	14	46.7	(28.3–65.7)
Bullous dermatoses	5	4	2	50.0	(6.8–93.2)
Erythematous event	3	2	1	50.0	(1.3–98.7)
Urticaria or rash	37	25	11	44.0	(24.4–65.1)
Dermatology					
Overall	21	13	5	38.5	(13.9–68.4)
Bullous dermatoses	7	6	3	50.0	(11.8–88.2)
Erythematous event	3	1	0	0.0	(0.0–97.5)
Urticaria or rash	13	7	2	28.6	(3.7–71.0)
Family/general practice					
Overall	34	22	11	50.0	(28.2–71.8)
Bullous dermatoses	3	1	1	100	(2.5–100)
Erythematous event	1	1	1	100	(2.5–100)
Urticaria or rash	30	20	9	45.0	(23.1–68.5)
Other specialties^d^					
Overall	56	40	17	42.5	(27.0–59.1)
Bullous dermatoses	8	6	3	50.0	(11.8–88.2)
Erythematous event	5	3	1	33.3	(0.8–90.6)
Urticaria or rash	45	32	13	40.6	(23.7–59.4)
With inclusion of incidental cases^g^					
Overall	265^f^	184	137	74.5	(67.7–80.2)
Bullous dermatoses	6	3	1	33.3	(0–70.8)
Erythematous event	15	9	6	66.7	(35.4–87.9)
Urticaria or rash	247	173	131	75.7	(68.8–81.5)

*Abbreviations*: *PMO* Post-Menopausal Osteoporosis, *PPV* positive predictive value, *CI* confidence interval

^a^Number of confirmed cases divided by number of obtained charts

^b^CI calculated using binomial exact method

^c^Treating providers may have more than one specialties. Thus, each potential case may be counted under multiple provider specialties.

^d^Other outpatient specialties were grouped due to small sample sizes

^e^Included cases of medical record confirmed hypocalcemia which were listed in the record as a secondary reason for the emergency department or hospital visit

^f^Three potential dermatologic adverse events had qualifying codes for multiple dermatologic subtypes.

^g^Included cases of medical record confirmed dermatologic adverse events which were listed in the record as a secondary reason for the emergency department or hospital visit

Medical records were sought for a random 265 of 441 potential dermatologic adverse events identified from the database (6 bullous, 15 erythematous, 247 urticaria/rash and 3 with multiple codes); 184 (69%) charts were received (all had sufficient information for adjudication). The physician confirmed 128 as dermatologic events (PPV 69.6%, 95% CI 62.4–76.1%) (Table 2). PPVs varied across subtypes of dermatologic events (highest for urticaria/rash [PPV 70.5%, 95% CI 63.1–77.2%]) and healthcare setting (highest for ED [PPV 77.0%, 95% CI 69.4–83.5%]). Additionally, the PPV was higher for claims associated with emergency medicine (PPV 78.1%, 95% CI 70.2–84.7%) relative to other provider specialties. The inclusion of incidental dermatologic adverse events had little impact (PPV 74.5%, 95% CI 67.7–80.2%).

## Discussion

In this nationwide, observational study of women with PMO, the performance of diagnosis codes in identifying hypocalcemia and dermatologic adverse events from health insurance claims data varied across settings, and by provider specialty. Our definition of hypocalcemia (as the primary reason for obtaining ED or inpatient care) yielded a PPV of 40%, and for dermatologic adverse events, a PPV of 70%. The inclusion of incidental cases increased the PPV of hypocalcemia appreciably, suggesting that secondary hypocalcemia may frequently be recorded in the primary position on claims. Incidental cases were infrequent for dermatologic adverse events, possibly because these events generally represent the true primary reason for the patients’ care. With both outcomes, the diagnosis codes from ED claims were more accurate than inpatient claims. Serious hypocalcemia and dermatologic adverse events may be treated and resolved within the ED without requiring hospital admission, and if hospitalization does occur, these outcomes—hypocalcemia in particular—may be considered a secondary concern.

There are few published data for comparison. Strom et al. reported that within Medicaid claims, 60.9% of the erythematous events captured through presence of ICD-9 695.1 (erythema multiforme, Stevens-Johnson syndrome, and toxic epidermal necrolysis) were later confirmed as true cases [15]. Within a health plan database, Chan et al. reported that the presence of a discharge diagnosis of erythema multiforme yielded a PPV of 60.7% [16]. These are similar to our PPV finding of 56 to 67% (including incidental cases) for serious erythematous events, which also included ICD-9 695.5 (exfoliation due to erythematous conditions).

Historically, 70 to 80% of medical records requested by our research group (and similar institutions) are obtained [17, 18]. In this study, as expected, our retrieval rate was at the lower end of this spectrum as we sought medical records only from the principal site of care. This choice arose from the study objective to validate outcomes associated with a specific medical claim, rather than to confirm the presence of an outcome. While our lower retrieval rate decreased the precision of the PPVs, leading to broader confidence intervals, it likely did not bias the PPVs estimates, unless the chart retrieval rate was somehow differential with respect to the true case status. For example, if hospitals were more likely to provide charts for true cases of hypocalcemia, our PPV estimates would be biased toward 100%. However, this scenario seems unlikely. With other study objectives, it is generally feasible to seek charts from multiple providers or institutions (e.g., a dermatologist and a hospital) to increase the fraction of events for which at least one medical record is available.

In this study, we had expected that limiting our algorithm to the first-position diagnoses on claims would increase the PPV for capturing serious occurrences of adverse events that were the primary reason for seeking care. However, we found that clinically incidental or secondary events are also captured through diagnosis codes recorded in the primary position. Further, it is important to note that outcomes leading to hospitalization or ED visits may have had *ICD-9* codes recorded in a secondary position on claims. These cases were not counted in this study, and thus, incidence derived with these code sets will be underestimated.

This study was conducted in a US commercially-insured population which, on average, tend to be slightly younger than the US general population. While we expect the results of this study to be generalizable to other insured populations, caution must be taken if there are differences in coding standards for reimbursement for hypocalcemia or dermatologic adverse events across insurers. Further, as PPVs vary according to disease prevalence, our PPVs may underestimate those observed in populations with a higher prevalence of hypocalcemia and/or dermatologic events than our study population, and overestimate those observed in populations with lower prevalence of these conditions than our study population. This highlights the need to assess the performance of case-identification algorithms within specific populations of interest. Lastly, we recognize that additional work is needed to assess the performance of algorithms for identifying other outcomes of interest that are associated with the use of osteoporosis medication, including osteonecrosis of the jaw, and atypical femur fractures.

## Conclusion

Our results suggest that the current algorithms to identify serious hypocalcemia and dermatologic adverse events are moderately accurate for events leading to an ED visit (PPV 81.8% for hypocalcemia and PPV 77.0% for dermatologic adverse events) and has lower accuracy for events leading to hospitalization. In certain scenarios, estimates derived from the current claims definitions may be insufficient, and algorithms that include other components of insurance claims data should be explored to further refine the algorithm. Alternatively, the outcome definitions could be widened to include all occurrences of the events that result in healthcare services.

## Additional file

Additional file 1: **Table S1.** Algorithm for identifying post-menopausal osteoporosis. (DOCX 14 kb)

## Abbreviations

CI: Confidence interval
ED: Emergency department
ORD: Optum Research Database
PMO: Postmenopausal osteoporosis
PPVs: Positive predictive values

## References

[1] US Department of Health and Human Services (2004). Bone health and osteoporosis: a report of the surgeon general.

[2] Lewiecki EM (2008). Denosumab: an investigational drug for the management of postmenopausal osteoporosis. Biologics.

[3] Papapoulos SE, Rosen CJ, Glowacki J, Bilezikian JP (1999). Bisphosphonates. The aging skeleton.

[4] Peter R, Mishra V, Fraser WD (2004). Severe hypocalcaemia after being given intravenous bisphosphonate. BMJ.

[5] Schussheim DH, Jacobs TP, Silverberg SJ (1999). Hypocalcemia associated with alendronate. Ann Intern Med.

[6] Phillips E, Knowles S, Weber E, Shear NH (1998). Skin reactions associated with bisphosphonates: a report of 3 cases and an approach to management. Allergy Clin Immunol.

[7] Brinkmeier T, Kügler K, Lepoittevin JP, Frosch PJ (2007). Adverse cutaneous drug reaction to alendronate. Contact Dermatitis.

[8] Loughlin J, Mast TC, Doherty MC, Wang FT, Wong J, Seeger JD (2012). Postmarketing evaluation of the short-term safety of the pentavalent rotavirus vaccine. Pediatr Infect Dis J.

[9] Schneeweiss S, Seeger JD, Landon J, Walker AM (2008). Aprotinin during coronary-artery bypass grafting and risk of death. N Engl J Med.

[10] Enger C, Gately R, Ming EE, Niemcryk SJ, Williams L, McAfee AT (2010). Pharmacoepidemiology safety study of fibrate and statin concomitant therapy. Am J Cardiol.

[11] Seeger JD, Loughlin J, Eng PM, Clifford CR, Cutone J, Walker AM (2007). Risk of thromboembolism in women taking ethinylestradiol/drospirenone and other oral contraceptives. Obstet Gynecol.

[12] Xue F, Ma H, Stehman-Breen C, Haller C, Katz L (2013). Design and methods of a postmarketing pharmacoepidemiology study assessing long-term safety of Prolia® (denosumab) for the treatment of postmenopausal osteoporosis. Pharmacoepidemiol Drug Saf.

[13] Centers for Medicare & Medicaid Services. Medicare claims processing manual chapter 23–fee schedule administration and coding requirements. [Accessed 17 Nov 2017]. Available at: https://www.cms.gov/Regulations-and-Guidance/Guidance/Manuals/downloads/clm104c23.pdf.

[14] Wilson EB (1927). Probable inference, the law of succession, and statistical inference. J Am Stat Assoc.

[15] Strom BL, Carson JL, Halpern AC (1991). Using a claims database to investigate drug-induced Stevens-Johnson syndrome. Stat Med.

[16] Chan H-L, Stern RS, Arndt KA (1990). The incidence of erythema multiforme, Stevens-Johnson syndrome, and toxic epidermal necrolysis. A population-based study with particular reference to reactions caused by drugs among outpatients. Arch Dermatol.

[17] Johannes CB, Ziyadeh N, Seeger JD, Tucker E, Reiter C, Faich G (2007). Incidence of allergic reactions associated with antibiotic use in a large managed care organization. Drug Saf.

[18] Seeger JD, West WA, Fife D, Noel GJ, Johnson LN, Walker AM (2006). Achilles tendon rupture and its association with fluoroquinolone antibiotics and other potential risk factors in a managed care population. Pharmacoepidemiol Drug Saf.

