# Supplementary material for: Predictive values of diagnostic codes for identifying serious hypocalcemia and dermatologic adverse events among women with postmenopausal osteoporosis in a commercial health plan database
Source: BMC Health Serv Res. 2018 Apr 10;18:263. doi: 10.1186/s12913-018-3016-y (PMC5891890; doi:10.1186/s12913-018-3016-y)
Supplement: Supplementary file 1 — Table S1. Algorithm for identifying post-menopausal osteoporosis. (DOCX 14 kb) [file 12913_2018_3016_MOESM1_ESM.docx]

Additional file 1: Table S1 Algorithm for identifying post-menopausal osteoporosis

| **Osteoporosis Diagnosis:** Osteoporosis (ICD-9 733.xx) associated with an inpatient or outpatient claim *OR*  **Osteoporosis Medication:** Dispensing or administration of an osteoporosis medication (brand name or generic) relating to the following generic names: alendronate, calcitonin, ibandronate, pamidronate, raloxifene, risedronate, teripratide, or zoledronic acid.  ***OR***  Osteoporotic Fracture: Fracture associated with osteoporosis, including a closed fracture of the hip, spine, pelvis, humerus, femur, or radius/ulna (ICD-9 733.11, 733.13, 733.15, 805.0x, 805.2x, 805.4x, 805.8x, 808.xx, 812.xx, 813.xx, 820.0x, 820.2x, 820.8x, 821.xx) associated with an inpatient or outpatient claim, or accompanied by a surgical repair code for the specific type of fracture |
| --- |

Abbreviation: ICD-9 International Classification of Diseases, Ninth Edition
